# Frequency tagging with infants: The visual oddball paradigm

**DOI:** 10.3389/fpsyg.2022.1015611

**Published:** 2022-11-08

**Authors:** Stefanie Peykarjou

**Affiliations:** Department of Psychology, Heidelberg University, Heidelberg, Germany

**Keywords:** frequency tagging, fast periodic visual stimulation, categorization, analysis strategies, visual processing, infants

## Abstract

Combining frequency tagging with electroencephalography (EEG) provides excellent opportunities for developmental research and is increasingly employed as a powerful tool in cognitive neuroscience within the last decade. In particular, the visual oddball paradigm has been employed to elucidate face and object categorization and intermodal influences on visual perception. Still, EEG research with infants poses special challenges that require consideration and adaptations of analyses. These challenges include limits to attentional capacity, variation in looking times, and presence of artefacts in the EEG signal. Moreover, potential differences between age-groups must be carefully evaluated. This manuscript evaluates challenges theoretically and empirically by (1) a systematic review of frequency tagging studies employing the oddball paradigm and (2) combining and re-analyzing data from seven-month-old infants (*N* = 124, 59 females) collected in a categorization task with artifical, unfamiliar stimuli. Specifically, different criteria for sequence retention and selection of harmonics, the influence of bins considered for baseline correction and the relation between fast periodic visual stimulation (FPVS) responses and looking time are analyzed. Overall, evidence indicates that analysis decisions should be tailored based on age-group to optimally capture the observed signal. Recommendations for infant frequency tagging studies are developed to aid researchers in selecting appropriate stimulation and analysis strategies in future work.

## Introduction

Stimulating the brain rhythmically and measuring the resulting rhythmic changes of electrical activity on the scalp has been successfully employed in vision research for many decades (Adrian and Matthews, 1934; Adrian, 1944; Walter et al., 1946; Dawson, 1954). More recently, rhythmic stimulation has been applied in cognitive research and provided evidence for very fast face and object categorization in infants and adults (Liu-Shuang et al., 2014; de Heering and Rossion, 2015; Rossion et al., 2015; Peykarjou et al., 2017; Kabdebon et al., 2022). Due to its objectivity and high signal-to-noise ratio, this approach holds great potential for developmental cognitive neuroscience. In particular, the so-called fast periodic visual stimulation (FPVS) oddball paradigm (Heinrich et al., 2009; Liu-Shuang et al., 2014) embeds categorical changes at a slower frequency rate into a fast periodic stream of stimuli and thus allows for dissociating the rate of categorization from low-level changes within a single stimulation stream. This paper summarizes studies utilizing the approach, addresses its methodological strengths and challenges and systematically examines analysis decisions on response patterns to the visual FPVS oddball paradigm in infants.

In the past, inducing rhythmic responses through visual stimulation has been described in the frameworks of steady-state visual evoked potentials (SSVEPs; Regan, 1966, Norcia et al., 2015), fast periodic visual stimulation responses (FPVS; Liu-Shuang et al., 2014, Rossion et al., 2020), and frequency tagging (Tononi et al., 1998). Here, the term frequency tagging will be adopted as it captures the idea of associating a cognitive process with a frequency tag, and thus stresses an important advantage of the approach, the *a priori* defined dependent measure. Frequency tagging can be understood as the process of stimulating in a given frequency and analyzing EEG responses at the corresponding frequencies. To this purpose, the continuous EEG signal recorded in the time-domain is converted to the frequency domain *via* a fast Fourier Transformation (FFT). Thus, changes in amplitude over time are converted and represented as amplitude and phase across frequencies, allowing the researcher to track response strengths at stimulated frequencies.

The frequency tagging approach has recently been employed to measure face individuation and object categorization with great validity, objectivity, reliability and sensitivity (Stothart et al., 2017; Rossion et al., 2020; Peykarjou et al., under review^1^). It is objective in the sense that the response of interest is defined *a priori* by the input frequency *f* (fundamental frequency), and analysis are confined to the input frequency and its harmonics (n*f*). Satisfactory reliabilities of adult frequency-tagging responses in face individuation have recently been established (Dzhelyova et al., 2019; Stacchi et al., 2019), but reliability certainly depends on the specific stimuli and design as well as processing proficiency of participants. It is conceivable that more variable responses in less capable participants will lead to decreased reliability (see also Stacchi et al., 2019). There also is first evidence that frequency tagging responses are correlated with performance, e.g., decreased face individuation responses in prosopagnosia (Liu-Shuang et al., 2016). In addition, previous work has demonstrated high sensitivity of frequency tagging, requiring only a few minutes of stimulation to reliably differentiate responses from noise (Dzhelyova et al., 2019). Finally, flickering images are highly attractive for infants, thus eliciting prolonged looking and alertness, further enhancing the usability of this approach in development.

Thus, frequency tagging holds great potential for developmental cognitive neuroscience, and has been successfully employed in vision and development over decades. Since the 1970, it was leveraged to delineate the development of spectral sensitivity, acuity, contrast sensitivity and binocular interaction (Dobson, 1976; Sokol, 1978; Atkinson et al., 1979; Petrig et al., 1981). During the last years, frequency tagging has received surging interest and been applied in face and object categorization, intermodal perception, and attention (De Heering and Rossion, 2015; Peykarjou et al., 2017; Barry-Anwar et al., 2018; Christodoulou et al., 2018; Leleu et al., 2020; Rekow et al., 2021). The present study systematically evaluates design and analysis decisions to develop recommendations for future applications of this method.

First, methodological differences between infant and adult studies will be described, focusing on particulars of frequency tagging (Hoehl and Wahl, 2012, for more general discussions of infant EEG studies, see DeHaan, 2007). This part is supplemented by a systematic review of existing studies following the FPVS oddball approach. In the empirical part, the effects of methodological decisions will be assessed.

### Methodological characteristics of frequency tagging employed with infants

A great advantage of EEG frequency tagging studies is that the same stimulation and dependent measures can be employed across age. As the frequency of interest is defined and embedded into the stimulation *a priori* by the researcher, responses at this precise frequency can be analyzed regardless of age-group or other sample characteristics. While one might adapt the speed of stimulation and thus the fundamental frequency for different samples, past research has employed the same speed for infants and adults and found that infants are capable of processing images at presentation times employed with adults, typically less than 170 ms per image. Anyhow, studies determining how infants respond to stimulation at different fundamental frequencies are lacking (see, for example, Gentile and Rossion, 2014; Retter et al., 2020, for evidence in adults) and would be very helpful for furthering our understanding of differences and commonalities in rhythmic responses throughout development.

**Figure 1:**
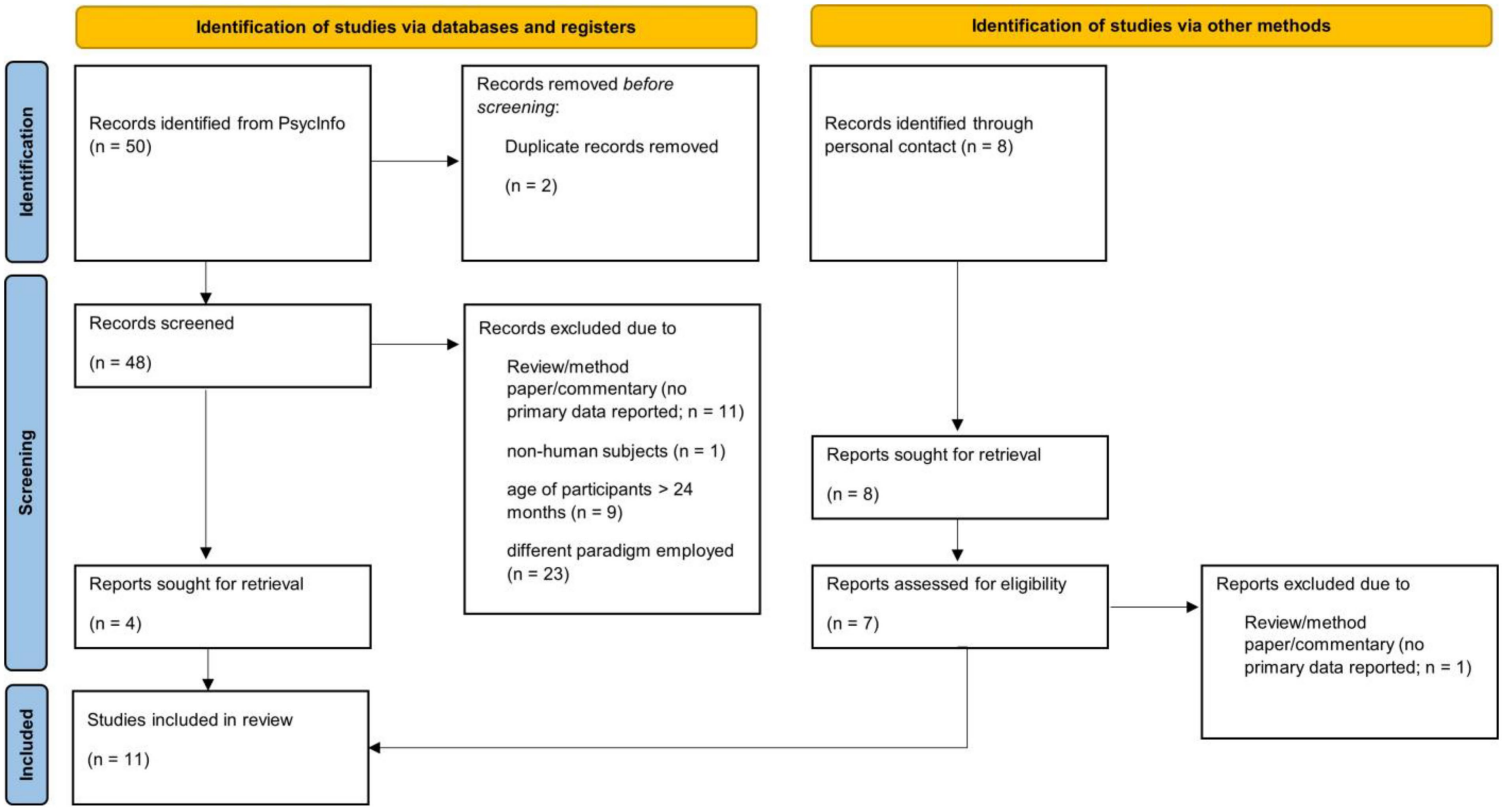
Flow diagram of study selection process for systematic review (Page et al., 2021).

While the general stimulation can thus be applied irrespective of age, some specifics require adaptation for infant participants. For example, infants’ attentional capacity is relatively short, which requires adapting the stimulation in terms of duration of flicker sequences, the number of sequences and, thus, the number of conditions that can be tested within-subjects. Moreover, the rather high amount of artefacts typically present in infant EEG requires careful handling. To provide an empirical overview of methodological decisions made in FPVS oddball studies with infants, a focused systematic review was conducted based on the search terms “infant” and “frequency tagging,” “SSVEPs” or “fast periodic visual stimulation” (see Figure 1).

The Psycinfo search was performed on March 23^rd^ 2022 and supplemented with records identified by personal communication. All studies employing a visual FPVS oddball paradigm with infant participants (< 4 months of age) were included, yielding *N* = 11 records (see Table 1 for an overview of all included studies). All information was double-coded by the author and student assistants. The following information was extracted from included articles, separately for all groups tested: mean participant age, sample size, software and hardware employed, number of within-subject conditions, duration of sequences, number of sequences presented and kept, criteria for excluding sequences, methods employed for artifact detection, treatment of artefacts, number of harmonics analyzed, criteria for selection of harmonics, number of bins for baseline-correction, and response strength of significant responses (see Tables 1, 2).

**Table 1:** Design of studies included in the systematic review.

First Author/Year of Publication	Journal	*N/M* age (mo)	Software/Hardware	No within – subject – conditions	Sequence duration /total presentation duration (s)
Baccolo et al., in preparation (see footnote 3)	submitted	29/6	24 inch, 60 Hz monitor/Matlab	2	15/120
Barry-Anwar et al., 2018	Neuropsychologia	23/619/9	Not specified/Matlab	2	10/200
Bertels, et al., 2020	Scientific Reports	39/8	800 × 600 monito, 60 Hz/Windows, Matlab, Psychotoolbox	2	20/251
De Heering and Rossion, 2015	eLIFE	30/5	not specified/Windows, Matlab, Psychtoolbox	1	20/160
Leleu et al., 2020	Developmental Psychology	18/4	24 inch, 60 Hz monitor/custom Java software	2	31.17/264.95
Peykarjou et al., 2017	Scientific Reports	41/9	60 Hz monitor/Windows, Matlab	2	20/212.1
Peykarjou et al., 2022	Cortex	39/5	60 Hz monitor/Windows, custom Java software	2	20/113.2
Peykarjou et al., under revision (see footnote 1)	under revision	44/4 40/756/11	60 Hz monitor/Windows, Matlab and custom Java software	1	20/166
Poncet et al., 2022	Frontiers in Neuroscience	18/3.5 18/7	24 inch, 60 Hz monitor/not specified	3	31.17/311.7
Rekow et al., 2020	Cognitive Development	18/4	24 inch, 60 Hz monitor/custom Java software	2	31.167/311.7
Rekow et al., 2021	Proceedings of the National Academy of Sciences	20/4	24 inch, 60 Hz monitor/not specified	2	31.17/280.53

**Table 2:** Analysis decisions of studies included in the systematic review.

First Author/Year of Publication	*M* no sequences pres./incl.	Criterion for sequence exclusion	Artifact detection/criterion for harmonics selection	Handling of artefacts	Bins for baseline-correction	Response strength (of significant categorization)	Harmonics considered (categorization)
Baccolo et al., in preparation (see footnote 3)	8/7.65	looking at screen for less than majority of sequence, SNR < 2 at base frequency or first harmonic on 70, 75 or 83 (=O1, O2, or Oz)	visual inspection/ Bonferroni-corrected Z-scores of grand-averages, averaged across conditions	interpolation, exclusion	10 (excl. Adjacent bins)	SNR 2.34	3rd to 4th harmonic summed
Barry-Anwar et al., 2018	20/11.70 20/9.79	looking at screen for <50%, artefacts/noise >50% of time	visual inspection/no harmonic responses evident	exclusion or values replaced with zeros	10 (excl. Adjacent bins)	SNR 1.05–1.27	no harmonic responses considered
Bertels, et al., 2020	12.55/8.41	looking at screen for less than majority of sequence, SNR < 2 at base frequency on O1, O2, and Oz	visual inspection/ based on literature	interpolation, exclusion, normalization of Fourier coefficients	10 (excl. Adjacent bins)	SNR 1.84–4.17	first 4 harmonics averaged
de Heering and Rossion, 2015	8/4.75	looking at screen for less than majority of sequence, SNR < 2 at base frequency on O1, O2, or Oz	visual inspection/ X	interpolation, exclusion	20 (excluding adjacent bins)	SNR 0.99–2.56	harmonics 1 and 2 described
Leleu et al., 2020	8.5/7.72	crying, > = 2 Z-scores >1.64 or 1 Z-score > 2.3 at base frequency and 1st harmonic on Oz, POz, O1, or O2	Artifact Blocking algorithm, visual inspection/Z-scores of grand-averages, averaged across conditions	Artifact Blocking algorithm, interpolation, exclusion	10	Z 1.91–4.79	only 1st harmonic significant
Peykarjou et al., 2017	10.60/9.25	significant response at base frequency, looking at screen for <50% of time	visual inspection/ Bonferroni-corrected Z-scores of grand-averages, averaged across conditions	interpolation, exclusion	10 (excl. Adjacent bins)	SNR 1.08–1.59	1st to 11th harmonic summed
Peykarjou et al., 2022	5.66/2.08	> 3 channels requiring interpolation, too many artifacts, SNR < 2 at base frequency and 1st harmonic on O1, O2, and Oz	visual inspection/ Bonferroni-corrected Z-scores of grand-averages, averaged across conditions	interpolation, exclusion	10 (excl. Adjacent bins)	BCA-43-1.82	1st to 9th harmonic summed
Peykarjou et al., under revision (see footnote 1)	6.68/3.59 8.46/5.27 9.28/5.14	> 3 channels requiring interpolation, too many artifacts, SNR < 2 at base frequency and 1st harmonic on O1, O2, and Oz	visual inspection/ Bonferroni-corrected Z-scores of grand-averages, averaged across conditions	interpolation, exclusion	20 (excl. Adjacent bins, and two extreme bins)	BCA 0.08–0.53	harmonics summed; 4 mo: 3rd harmonic, 7 ms 3rd - 4th harmonic, 11 mo 2nd - 4th harmonic
Poncet et al., 2022	<=14/10.11 < =14/10.56	> = 2 Z-scores >1.64 or 1 Z-score > 2.3 at base frequency and 1st harmonic on Oz, POz, O1, or O2; global noise-corrected amplitude > < 2 SD of mean of all sequences, < 2 sequences left after preprocessing	Artifact Blocking algorithm, visual inspection/Z-scores of grand-averages	Artifact Blocking algorithm, interpolation, exclusion	10	SNR 2.05–4.34	condition 1: only stimulation frequency significant, condition 2: 1st to 2nd harmonic summed
Rekow et al., 2020	10/9.20	> = 2 Z-scores >1.64 or 1 Z-score > 2.3 at base frequency and 1st harmonic on Oz, POz, O1, or O2; global noise-corrected amplitude > < 2 SD of mean of all sequences, only one sequence left after preprocessing	Artifact Blocking algorithm, visual inspection/Z-scores of grand-averages	Artifact Blocking algorithm, interpolation, exclusion	10	Z 2.44	1st to 3rd harmonic summed
Rekow et al., 2021	9/8.50	> = 2 Z-scores >1.64 or 1 Z-score > 2.3 at base frequency and 1st harmonic on Oz, POz, O1, or O2; global noise-corrected amplitude > < 2 SD of mean of all sequences	Artifact Blocking algorithm, visual inspection/Z-scores of grand-averages	Artifact Blocking algorithm, interpolation, exclusion	10	Z 1.76–3.13	only 1st harmonic significant
	SNR 1 channel >1.5	SNR 1 channel >2	SNR 2 channels >2	SNR 3 channels >2	Z-score 1 channel >2.33	Z-score 2 channels >1.64	Z-score 3 channels >1.64
*M* (SD) number of remaining sequences	5.76 (2.94)	5.18 (2.90)	4.39 (2.82)	2.96 (2.43)	5.02 (2.80)	4.75 (2.79)	3.54 (2.60)
*M* (SD) number of excluded sequences	0.06 (0.25)	0.64 (0.90)	1.43 (1.52)	2.86 (2.24)	0.80 (1.05)	1.07 (1.23)	2.28 (1.78)
*N* remaining participants	124	123	119	107	123	123	113
SNR of GA categorization response	1.32	1.32	1.37	1.40	1.35	1.37	1.33
SNR of GA base response	3.12	3.32	3.40	3.40	3.27	3.34	3.14
Correlation of 6 Hz response with looking time		0.39 (*N* = 546)	0.34 (*N* = 418)	0.29 (*N* = 292)	0.37 (*N* = 529)	0.35 (*N* = 498)	0.34 (*N* = 366)
BF of correlation 6 Hz + looking time		7.23e^17^	1.48e^10^	23,553	4.67e^15^	5.73e^12^	2.13e^8^

Comparison of exclusion rates, grand-average responses and relations with looking times across different criteria for sequence retention.

Due to infants’ limited attentional capacity, the duration of frequency tagging sequences is reduced compared to studies with older participants. The literature review (see Table 1) indicates that previous studies employed sequence durations of 15–30 s with infants, whereas 60 s are typical for adults (e.g., Liu-Shuang et al., 2014; Stothart et al., 2017; Stacchi et al., 2019). This adaptation allows researchers to provide more breaks, soothe infant participants if necessary, and redirect attention to the screen. However, by adapting the duration of sequences, the resulting frequency resolution is also changed as both are inversely related *via* the FFT (1/duration(s)). This leads to a lower frequency resolution when using shorter stimulation times with infants. Moreover, the duration of sequences is potentially related to signal strength at the tagged frequency for two reasons (e.g., Dzhelyova et al., 2019): First, increasing stimulation time may support perception of embedded rhythms, thus strengthening responses, and second, a higher frequency resolution may enhance the signal-to-noise ratio by confining the stimulated response to a narrow frequency bin relative to the broad-band EEG noise.

In addition to reducing stimulation time, infants’ attentional span also limits the number of conditions that can be tested within-subjects. Based on the systematic review, infants (0–11 months) on average watched *M* = 213.06 (SD = 89.51) seconds of stimulation, arranged into 1–3 conditions. Most common were sequence durations of 15–30 s, so that an overall average of *M* = 9.91 (SD = 3.60) sequences were presented. Considering that some sequences will need to be excluded during preprocessing (see below), in most cases, running two to three conditions within-subjects is feasible.

Another caveat posed by infants’ attentional limits is variable looking time. Infants cannot be instructed to attend to the screen and will thus engage and disengage in a variable pattern during frequency tagging sequences. In contrast, most adult studies employ an orthogonal task to control attention and keep it constant (Liu-Shuang et al., 2014; Stothart et al., 2017; Stacchi et al., 2019). When participants look away from the screen, this may reduce the response strength as, on the one hand, they do not observe the stimulation and, on the other hand, they likely produce motor artefacts. Faced with this problem, all infant studies define a minimal threshold for inclusion of sequences, mostly based on responses to the general visual stimulation, sometimes considering looking times and/or data quality criteria (see Table 1 for an overview of inclusion criteria). Determining a criterion for retention of sequences is critical. On the one hand, sequences that were not attended well should be excluded, as categorization responses cannot be measured validly. On the other hand, as much data as possible should be retained to remain representative of the original dataset.

As elaborately discussed elsewhere (DeHaan, 2007; Hoehl and Wahl, 2012), infant EEG is characterized by a high amount of artefacts. These can be due to movement, limited preparation time during capping (leading to higher impedances), sweating or biological processes (e.g., heartbeat or respiration). Artefacts impact frequency tagging responses differently than other dependent variables, such as event-related-potentials (ERPs), as they are distributed broadly but not necessarily evenly in the frequency spectrum. When the infant blinks, she will do so in a partly random fashion, thus contributing to a range of frequencies. This means that artefacts may distort amplitudes across ranges of frequencies rather than at specific frequencies.^2^ Analyses of responses spread across the frequency spectrum, such as ERPs, are heavily influenced by these artefacts. In contrast, frequency tagging is based on analyzing specific frequencies of interest, and while artefacts may influence amplitude at each specific frequency, they will exhibit a similar influence on surrounding frequency bins and can thus be controlled by baseline correction.

The artefacts distributed throughout the spectrum increase standard deviations (SDs) of amplitudes across frequencies. This increased SD of whole frequency ranges may diminish the strength of the baseline corrected signal compared to cleaner data collected with older participants. Therefore, artefacts will enhance variability of the signal across bins, likely diminishing the statistical significance of responses at frequencies of interest. However, they will not influence the tagged frequency specifically, providing one major strength of this approach.

Dealing with artefacts is a central aspect of EEG analysis. Similar to other EEG approaches, in the context of frequency tagging, artefacts can be identified visually or by algorithms. As can be seen in Table 2, previous studies treated artifacts most commonly by excluding data, interpolating channels, and applying an artifact blocking algorithm. Semi-automizing the detection and handling of artifacts seems an important venture for the near future to facilitate and objectify analysis.

To determine the strength of the response at the tagged frequency, amplitude at this frequency is compared to and corrected for amplitude at the surrounding bins (baseline correction). The systematic review indicates that 10 bins (5 on each side, excluding the immediately adjacent bins), corresponding to approx. + − 0.35 Hz, are employed most frequently. The number of bins taken into account is reduced relative to typical studies with adults (considering 20 bins, Liu-Shuang et al., 2014; Stacchi et al., 2019) due to the shorter duration of sequences and, in turn, lower frequency resolution. Thus, by reducing the number of bins, a similar frequency range is taken into account as in longer sequences.

Tagged responses are analyzed at the stimulation frequency (*f*) and its harmonics, multiple integers of the stimulation frequency (n*f*). Due to non-linear properties of the stimulation and brain processes, responses can be spread across the stimulation frequency and harmonics, and thus the signal needs to be aggregated to provide a balanced comparison of conditions (Norcia et al., 2015; Retter et al., 2021). The systematic review reveals that recent studies statistically evaluate the presence of harmonics and proceed by summing significant harmonics, consistent with current recommendations (Retter et al., 2021).

Previously, it has been demonstrated that the pattern of dominant harmonics (i.e., the harmonic in which the highest amplitude is elicited) is relatively stable in adult participants over the course of 2 months (Dzhelyova et al., 2019). Very little is known regarding potentially systematic relations of harmonics with age, and, more generally, the number of studies comparing responses across age are still limited. In studies on face categorization (De Heering and Rossion, 2015; Rossion et al., 2015), the number of harmonics was reduced in 4-to-6-month-old infants compared to adults. In infants, only the first harmonic (i.e., the stimulation frequency) reached significance, whereas harmonics 1 to 14 were significant in adults. In a study on categorization of animals and furniture items, the number of significant harmonics increased from one to three harmonics during infancy (four to 11 months), compared to significant harmonics 1 to 22 in adults (Peykarjou et al., under review see footnote 1). In a similar vein, a study on facial trustworthiness discrimination observed harmonics 2 to 7 to be significant in adults, whereas only two harmonics reached significance in six-month-old infants (Baccolo et al., in preparation see footnote^3^). Thus, though more systematic work is needed, it appears that responses are confined to fewer harmonics during infancy than in adulthood.

In general, whether and how many harmonics are elicited varies much between tasks and samples, and it is currently still unclear which factors drive these differences. Importantly, as harmonics may differ between conditions and samples, they need to be considered during analysis to provide an unbiased estimation of responses elicited. If harmonics are present, they can unequivocally be attributed to the stimulation, as no enhanced harmonic responses are elicited when there is no stimulation frequency *f*.^4^ Some frequency tagging papers have passed over the analysis of harmonics (e.g., Barry-Anwar et al., 2018; Bekhtereva et al., 2018), which may lead to biased results. There may be cases where the first harmonic does not reach significance (e.g., Peykarjou et al., 2022), so focusing exclusively on the first harmonic would lead to the erroneous conclusion that no frequency tagging response was observed. On the other hand, even if the fundamental frequency is present, when conditions vary regarding the recruitment of harmonics, employing only the first harmonic would bias comparisons of conditions. Thus, inspection and balanced inclusion of harmonics is recommended, for example by averaging across conditions and extracting significant harmonics based on this overall grand-average.

Another decision that is potentially influenced by age (or other sample characteristics) is selecting regions of interest (ROIs). Based on the existing evidence, it seems that general visual activation associated with the flicker stimulation can be recorded in infants and adults likewise over the medial occipital cortex, predominantly on electrode Oz and spreading to O1/O2 (e.g., Leleu et al., 2020; Rossion et al., 2020; Pauen and Peykarjou et al., under revision ^5^). This is independent of the type of visual stimuli employed, consistent with the assumption that this base response reflects general visual processing. Selecting appropriate ROIs for the specific cognitive processes involved in frequency tagging studies in an age-fair way is much more challenging. Drawing on the limited number of studies in which the same paradigm was conducted with infants and adults (Baccolo et al., in preparation see footnote 3; Peykarjou et al., under review see footnote 1; de Heering and Rossion, 2015; Rossion et al., 2015), it seems that categorization responses are often confined to smaller ROIs in infants than adults. Adult data may provide a reasonable starting point for determining the spatial layout of frequency tagging responses, but the response may be restricted to fewer electrodes early in development.

A final challenge in developmental frequency tagging studies pertains to the reliability of responses. First reports on reliability in frequency tagging with adults are highly promising, indicating that base and cognitive responses can be measured with high retest-reliability in adults (Dzhelyova et al., 2019). As low reliabilities may pose a challenge in ERP studies (Cassidy et al., 2012; Munsters et al., 2019), this finding is very promising and may provide a basis for individual diagnosis in frequency tagging. So far, there are no reports on reliability of frequency tagging in infants, but due to the limitations discussed already, it can be expected to be lower than in adults. Among other factors, reliability increased linearly with increasing stimulation time in adults (Dzhelyova et al., 2019), so the shorter stimulation time with infant samples may limit reliability.

To sum up, employing frequency tagging with infants holds great potential for cognitive research, but is associated with challenges regarding stimulation and analysis. The stimulation needs to be adjusted to accommodate infants’ attentional limits by decreasing duration of sequences and limiting the number of conditions. During analysis, artefacts need to be handled with care, and decisions on inclusion of harmonics and ROIs need to be made in an age-fair way.

In the following, the influence of analysis decisions on response strengths will be explored empirically, drawing upon a large dataset of categorization by seven-month-old infants in a simple oddball categorization task. The empirical part will focus on comparing different criteria for retaining sequences, evaluating the relation between looking time and EEG responses, the influence of the frequency range for baseline correction and the number of sequences per participants, as well as the influence of considering different numbers of harmonics.

## Analyses of infant frequency tagging data

### Stimuli and design

**Figure 2:**
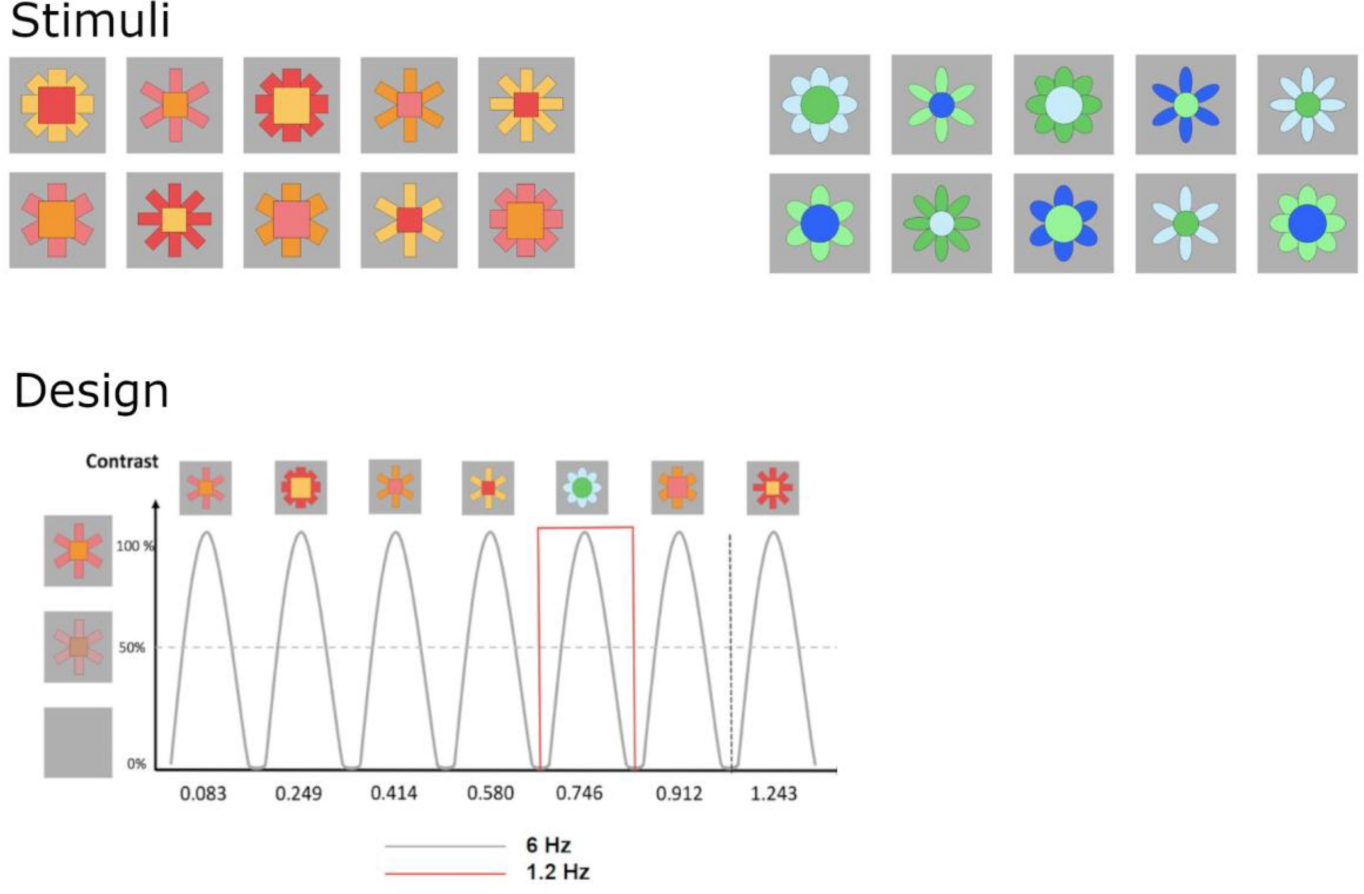
Schematic illustration of the stimuli and experimental paradigms. Ten different angular red-orange and round blue-green stimuli were employed, with contrasting categories differing in the shape of all parts (angular vs. round outlines) and colour spectrum. Phase-scrambled control images were created from original stimuli. In 20-s-sequences, images were presented by sinusoidal contrast modulation at a rate of 6 cycles per second = 6 Hz (1 cycle ≈ 170 ms). Angular red-orange stimuli (A) were presented as standards, with round blue-green stimuli presented as deviants at every 5^th^ cycle (B; 6/5 Hz = 1.2 Hz).

Infants were presented with sequences of artificial stimuli flickering at 6 Hz (6 items/s). At every fifth position, the type of stimulus changed, corresponding to 1.2 Hz (6/5 = 1.2, see Figure 2). Two types of stimuli were employed, red-orange/curvy shapes and blue-green/straight-edged shapes, with 10 individual exemplars of each type varying in size, color and number of pedals and parts (Pahnke, 2007; Ropeter and Pauen, 2013). Global luminance contrast did not differ across categories (*p* > 0.05). Pixel size of images was 545 (width) x 542 (height), corresponding to approximately 8 × 8 degrees of visual angle. Participants were compiled across two studies, so the maximum of sequences varied between 8 (Experiment 2) and 16 (Experiment 1).

Sequences were initiated manually when participants looked attentively at the screen and showed an artifact-free EEG signal. Each sequence started with a uniform grey background (random length, 5–6 s) while a short doorbell-sound was played to attract attention if necessary. Each sequence lasted 24 s, consisting of a fade-in of 2 s (contrast of images against the background increased gradually from 0 to 100%), a stimulation sequence of 20 s, and a fade-out of 2 s. Fade-in and fade-out phases served to avoid surprise reactions and blinks. Images changed size (+/− 5%) and were presented using a sinusoidal contrast-modulation function. Testing ended when participants became too inattentive or fussy.

### Sample

A sample of *N* = 124 (mean age = 7 months, 14 days, SD = 9 days, 59 females) participants were aggregated across two studies. On average, participants participated in *M* = 6.91 sequences (SD = 2.60, range 1–14), of which *M* = 1.09 (SD = 1.43, range 0–6) were excluded due to bad data (see specifics in Recordings and Analyses). A data base of *M* = 5.82 sequences (SD = 2.94, range 1–12) was thus employed for all analyses. All data combined here was based on the same stimulation, a categorization task with artificial categories as described in the section on stimuli & design. The first study evaluated the impact of familiarization on categorization responses. Categorization in the frequency tagging paradigm was compared following three different familiarization conditions: an ERP familiarization with stimuli from the base category (10 different exemplars presented 5 times for 1 s each); familiarization to the base category with subsequent presentation of a stimulus from the contrasting category (10 different exemplars presented 5 times for 1 s each, one stimulus from contrasting category presented for 15 s), and no familiarization. A previous study indicated that categorization did not differ based on familiarization condition (Experiment 2 in Pauen and Peykarjou, under revision see footnote 5), so data from participants was collapsed here across familiarization conditions. In the second study, subjects participated twice (T1, T2) in the same categorization task with an interval of approximately 2 weeks (data partly reported in Exp. 1, Pauen and Peykarjou, under revision see footnote 5). Only data from T1 are included into the aggregated sample. 41 additional infants were tested but excluded due to excessive crying (Experiment 1 *N* = 2, Experiment 2 *N* = 2), fussiness (Experiment 1 *N* = 7, Experiment 2 *N* = 7), too many artefacts (Experiment 2 *N* = 18), or technical problems (Experiment 1 *N* = 2, Experiment 2 *N* = 3). In accordance with the terms provided by the local ethics committee of Heidelberg University that approved the general procedure, written informed consent was obtainted from caretakers.

### Procedure

In a dimly lit and quiet room, infants sat on the lap of their caregivers with a computer monitor in front of them (looking distance of approx. 80 cm). Parents were asked not to interact with their infant during data collection. Infant behavior was recorded on video, and brain responses were registered using a BrainProducts actiCap (Gilching, Germany) with 32 active Ag-AgCl electrodes arranged according to the 10-10-system and a right mastoid reference.

### Recordings and analyses

Sampling rate was set at 250 Hz and the EEG signal was amplified using BrainAmp. Impedances were considered acceptable if <20 kΩ. EEG processing steps was performed using Matlab 2012b (The Mathworks) and Letswave.^6^ Data was band-pass filtered at 0.1–100 Hz using a 4th order Butterworth filter and segmented for each sequence. Noisy channels were identified and pooled from surrounding channels (for a maximum of three channels) and a common average reference computation was applied to all channels.

Preprocessed data segments were cropped to an integer number of 6 Hz cycles beginning 5 s after onset of the sequence (after fade-in) until 20 s (120 cycles, 5,000 time bins in total = 20 s). Sequences were averaged per participant before a Fast Fourier Transform (FFT) was applied to these averaged segments to extract amplitude spectra for all channels (square root of sum of squares of the real and imaginary parts divided by the number of data points). By averaging prior to the FFT, the contribution of responses not in phase (predominantly noise) is reduced (Rossion et al., 2020). Frequency analysis yielded spectra with a high frequency resolution of 0.05 Hz (1/20s).

The utility of different criteria for retention of sequences was compared by evaluating the number of sequences and participants included by criterion, the relation of each criterion with looking time data and the resulting pattern of base and categorization data. Amplitude at the base frequency (6 Hz) and its harmonics represents the brain’s response to the general visual stimulation and has previously been used as an index of whether the infant paid attention to the screen (de Heering and Rossion, 2015; Peykarjou et al., 2017). However, as the base frequency is also a multiple integer of the categorization frequency (5*1.2 Hz), both general stimulation and categorization processes may contribute to it. Base rate criteria can be based on SNR or Z-scores and are generally evaluated on channels O1, O2, Oz at 6 and, in several studies, 12 Hz. Here, the following base rate criteria will be evaluated: On O1, O2 or Oz, for 6 or 12 Hz, at least 1 SNR > 1.5; 1, 2 or 3 SNR > 2; 1 Zscore > 2.33; 2 or 3 Z-scores > 1.64. All following steps employ the base rate criterion selected based on this comparison. EEG amplitude at the frequency of *F*/5 = 1.2 Hz and its harmonics (i.e., 2*F*/5 = 2.4 Hz, 3*F*/5 = 3.6 Hz…) served as an index for categorization. In accord with prior work and recommendations (e.g., Peykarjou et al., 2017; Retter et al., 2021), responses were summed across significant harmonics. The range of harmonics for quantification of responses was defined by extracting all harmonics above noise level at a *p* < 0.05threshold. This threshold was Bonferroni-corrected for the number of tests run, in this case three independent tests for electrodes O1, O2 and Oz. Harmonics 1–19 reached significance and were summed for the categorization response, and harmonics 1–5 for the base response.

Looking time was coded offline. Twenty percent of videos were independently double-coded by two student assistants to estimate reliability, which reached high levels, Cronbach’s alpha = 0.96. Based on these analyses, a base rate criterion was selected and employed for all subsequent analyses.

To measure the magnitude of activity at pre-defined bins of interest, baseline corrected amplitudes were computed by subtracting the average amplitude of surrounding bins. SNRs were computed by dividing the signal by the amplitude at the neighboring frequency bins and used to display response patterns. Similarly, Z-scores were calculated by subtracting the average baseline-corrected amplitude of surrounding bins from each bin and dividing this by the standard deviation (SD) of the surrounding bins. The number of bins employed for statistical analysis was compared for 10 and 20 bins (removing the 2 bins with highest and lowest amplitude), corresponding to a frequency range of + − 0.35 to + − 0.70 Hz (excluding the immediately adjacent bins). Based on these analyses, the number of bins included in all further analyses was selected.

As a next step, the number of sequences included was varied systematically from 1 to 9 to evaluate how base and categorization response strengths relate to the number of sequences. To explore the impact of considering harmonics on response strengths, the number of harmonics was then increased systematically from 1 to 29 for the categorization response (always excluding harmonics corresponding to the base frequency, i.e., 5, 10, 15, 20, 25), and from 1 to 10 for the base response. The resulting data pattern was compared to the analysis based on harmonics selected according to Z-scores. Based on prior work (Peykarjou et al., 2017) and visual inspection of grand averages, all analyses were focused on visual responses recorded at O1, O2, and Oz. For all comparisons of different approaches with approximately similar numbers of participants, Friedman tests, a non-parametric alternative for repeated-measures ANOVAs, were employed to test for statistical significance.

### Results

#### Evaluating different criteria for retention of sequences

Sequence inclusion criteria were evaluated first by comparing the numbers of sequences and participants kept by criterion, and in a second step by examining the resulting pattern of base and categorization data and calculating the relation of responses with looking time data. An overview of the results can be found in Table 2.

Across all criteria for retaining sequences, the same harmonics were summed. Inspection of Bonferroni-corrected Z-scores revealed that a significant base response was obtained across harmonics 1 through 5 regardless of sequence retention criterion, while the 6th harmonic failed to reach significance. A significant categorization response was observed across harmonics 1–19, with the following exceptions: In the analysis on 3 channels with an SNR > 2 and 1 channel with Z > 2.33, the 18^th^ harmonic failed to reach significance, and for 3 channels Z > 1.64, the 2^nd^ harmonic failed to reach significance. Vice versa, the 21^st^ harmonic reached significance only when considering 2 channels Z > 1.64. For consistency, the dominant pattern of significant harmonics 1–19 was included.

As revealed by Table 2, rates of sequence exclusion ranged from.06 to 2.86. On average, criteria which were focused on only one channel excluded less than one sequence per participant. Likewise, while participant exclusion rates ranged from 0 to 13.7%, no more than one participant was excluded when criteria focused on only one channel. In contrast, when 2 or 3 channels were considered, more than 1 sequence and up to 13.7% of participants were excluded.

Signal-to-noise-ratios (SNRs) of resulting response patterns varied only slightly across criteria. For the categorization response, variation of average SNRs was no more than 4%, and less than 5% for the base response. Regardless of which criterion was employed, a substantial but non-exhaustive correlation (*r* = 0.29–0.39) between the base rate response and looking times emerged.

Overall, sequence retention criteria varied regarding participant retention, but negligibly regarding response patterns. Thus, a more inclusive criterion seems desirable considering parsimony of data collection and representativeness of the obtained sample. Therefore, in accord with the majority of prior research as indicated by the systematic literature review (Table 1), SNR > 2 on at least one channel at 6 or 12 Hz was selected. All further analyses are based on sequences included according to this criterion.

#### Comparing the number of bins employed for baseline correction

**Figure 3:**
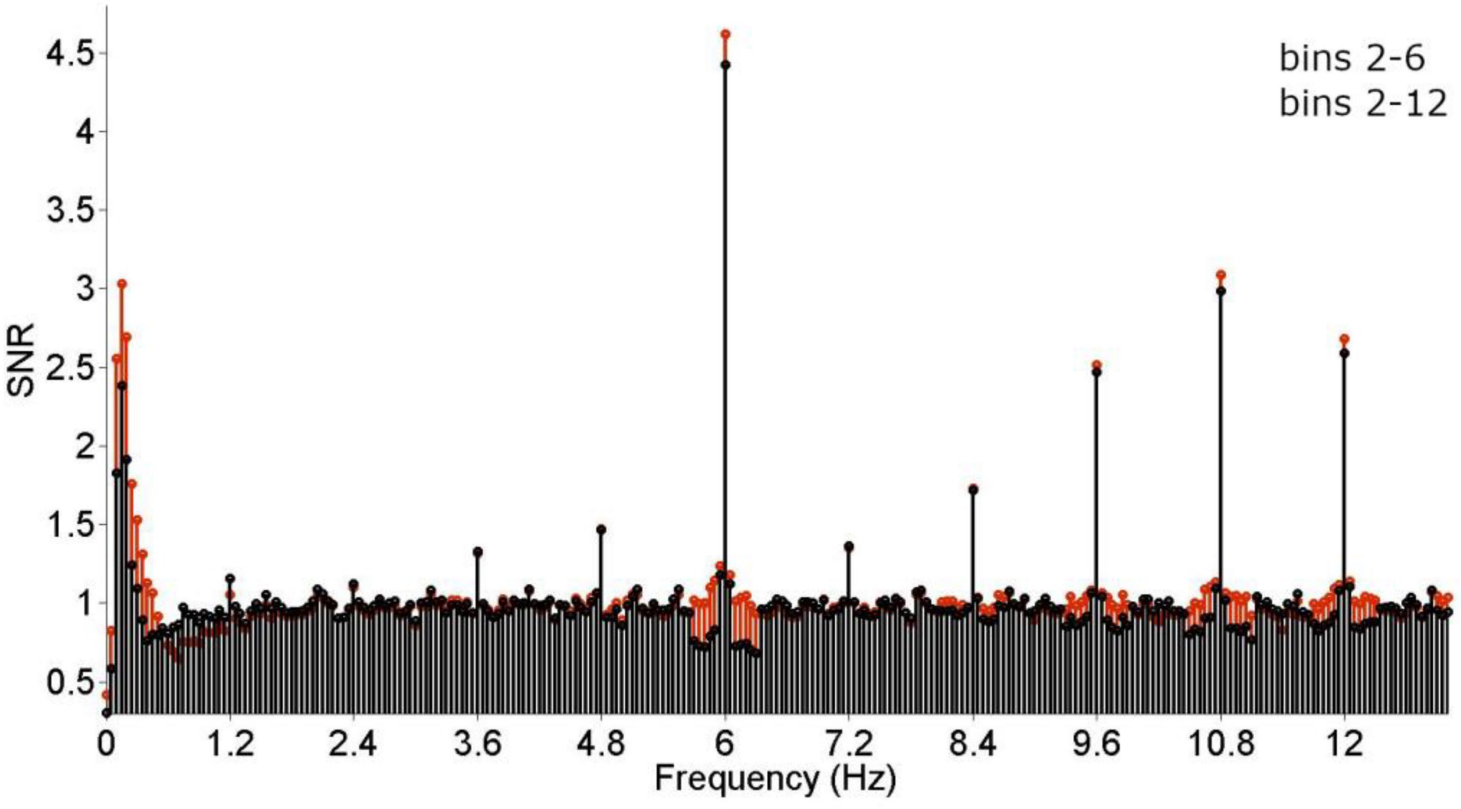
Grand-average frequency-spectrum responses. Base (6 + 12 Hz) and categorization (1.2, 2.4, 3.6… 10.8 Hz) responses are clearly visible and highly significant. The effect of the number of bins employed for baseline correction was evaluated by comparing signal-to-noise-ratios (SNRs) was compared across 10 (2–6) and 20 (2–12) bins. While employing a larger number of bins did not seem to affect responses at higher frequencies (from 6 Hz onwards) detrimentally, response strength was stronger when employing fewer bins at lower frequencies (below 6 Hz). Crucially, the effect seemed strongest at 1.2 Hz, corresponding to the stimulation frequency.

Next, the number of bins employed during baseline correction was compared. In adults watching 60-s-sequences, typically 20 bins are employed (Stothart et al., 2017; Rossion et al., 2020), whereas in infant studies, mostly 10 bins are used (see Table 1). These two criteria were evaluated by comparing the resulting grand-average SNRs and the results pattern of individual participants. As evident from Figure 3, while the use of 20 bins results in visually higher baseline-corrected amplitudes at higher harmonics of the categorization frequency, it decreases the response at 1.2 Hz. This is most likely due to the steep amplitude curve at the very low spectrum, which unproportionally skews the baseline correction at 1.2 Hz. Statistical tests including all significant harmonics (i.e., 1–19 for the categorization response, 1–5 for the base response) did not indicate an effect of the number of bins considered, *p* < 0.05.However, a hypothesis-driven analysis on 1.2 Hz only revealed that the response at this frequency is indeed reduced when employing 10 bins, *X^2^*(123, *N* = 123) = 25.609, *p* < 0.001. Considering that measuring at the stimulation frequency 1.2 Hz is essential to this paradigm, employing 10 bins is recommended for studies using shorter presentation times (<= 30 s).

#### Systematically increasing the number of included sequences 1 to 9

The number of sequences included was increased systematically from 1 to 9. Naturally, the number of participants contributing to the analysis decreased with increasing numbers of required sequences. Thus, analysis was stopped at *i*_sequences_ = 9 as the number of participants providing larger numbers of sequences was too small (*N* < 10).

**Figure 4:**
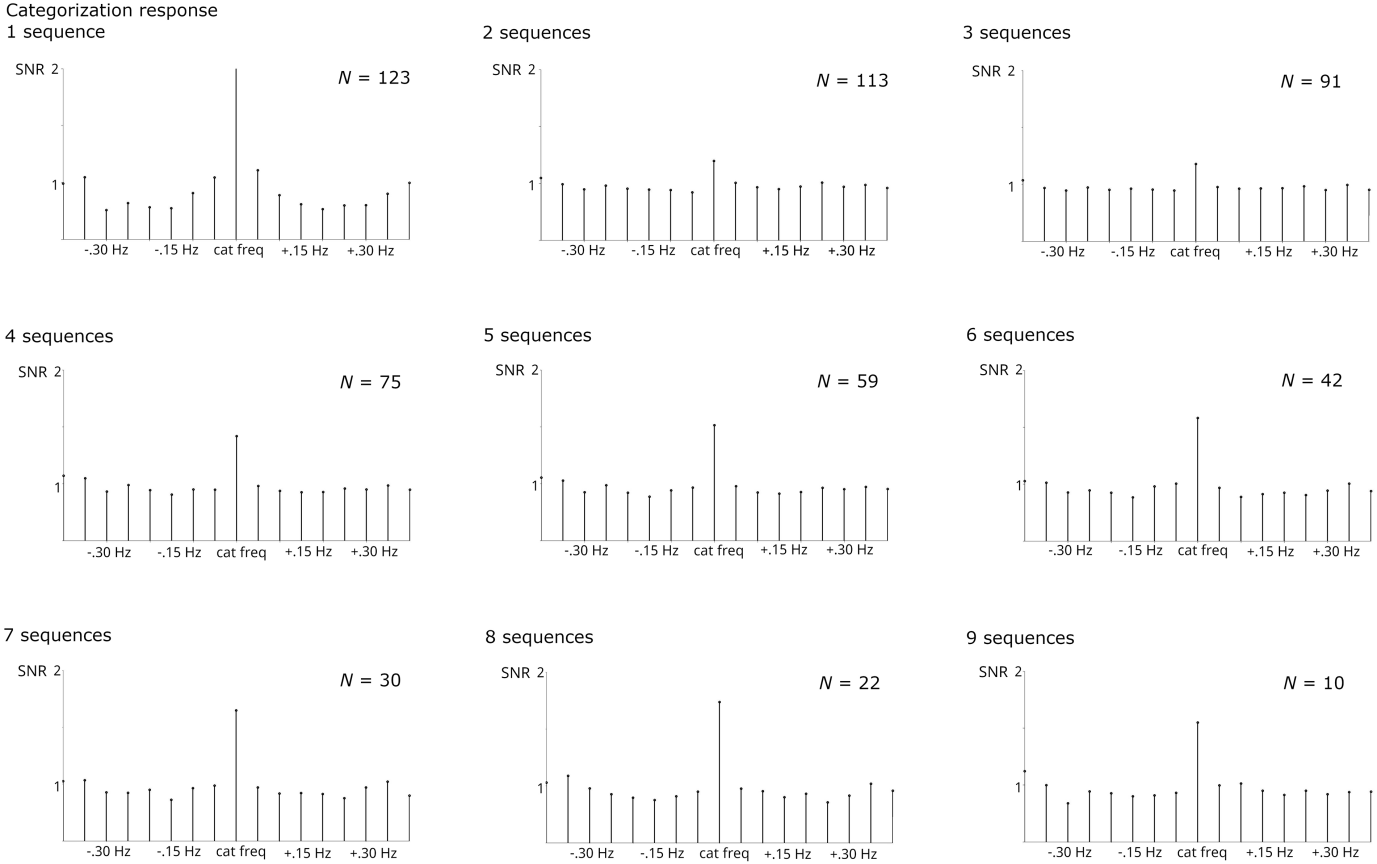
SNR of grand-average base responses summed across harmonics 1–5 and compared across numbers of sequences averaged. The respective *N* contributing to each analysis is specified per graph. Highly significant base responses were obtained in all analyses.

**Figure 5:**
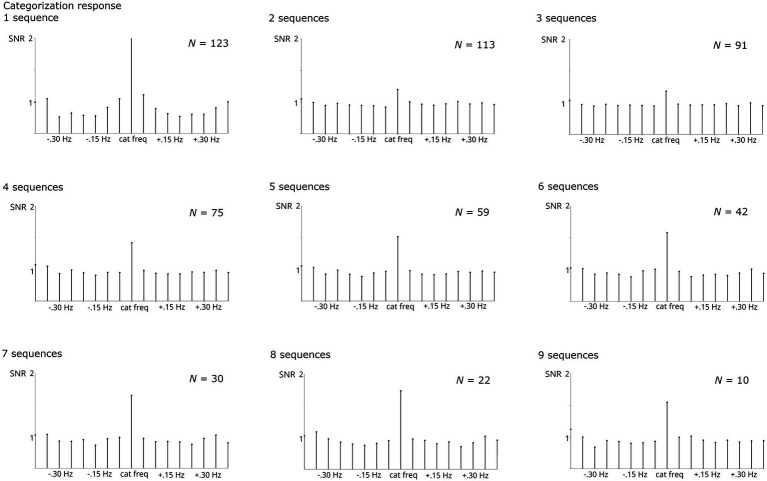
SNR of grand-average categorization responses summed across harmonics 1–19 and compared across numbers of sequences averaged. The respective *N* contributing to each analysis is specified per graph. Highly significant categorization responses were obtained in all analyses.

Regardless of the number of sequences considered, all analyses revealed significant responses, Zs > 11. Numerically, the base response was smaller when averaging across <4 sequences compared to higher numbers (Figure 4). As evident from Figure 5, the categorization response was numerically lower when averaging across 2–3 sequences. Importantly, responses were comparable when taking into account all children providing at least one sequence and higher sequence numbers. This descriptive analysis was not supplemented by inferential statistics as the number of subjects contributing to the different conditions varies substantially, violating assumptions of statistical tests.

**Figure 6:**
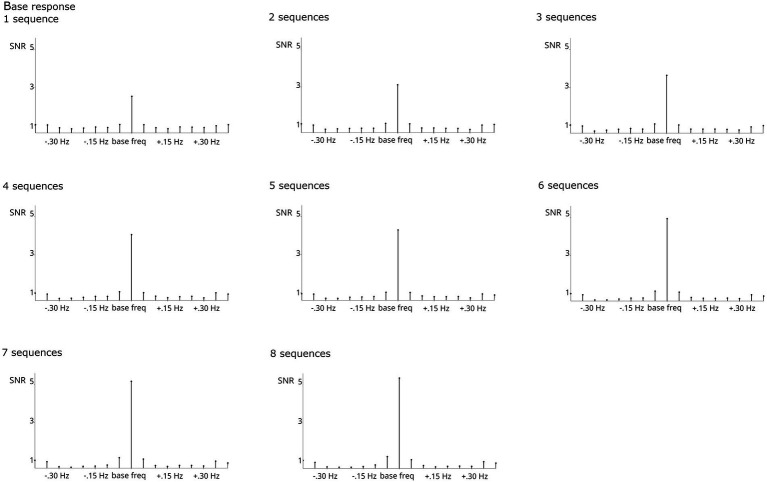
SNR of grand-average base responses summed across harmonics 1–5 and compared across numbers of sequences averaged for a subsample of *N* = 20 who provided usable data for at least 8 sequences. Highly significant base responses were obtained in all analyses, with a tendency for increasing amplitude with number of sequences averaged.

**Figure 7:**
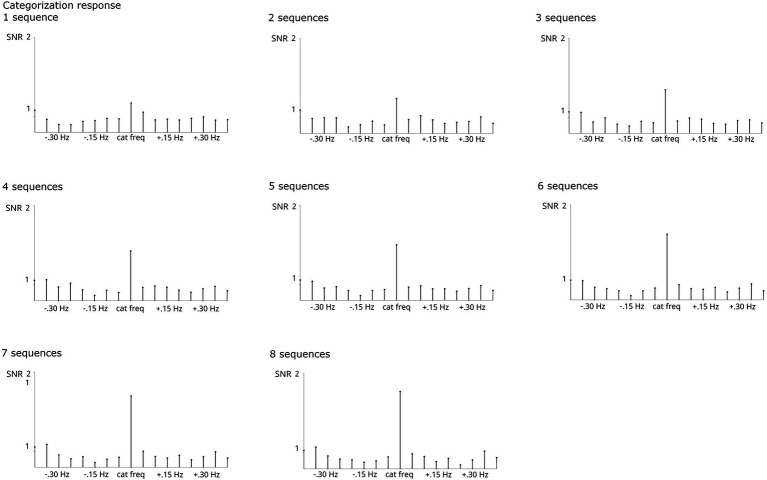
SNR of grand-average categorization responses summed across harmonics 1–19 and compared across numbers of sequences averaged for a subsample of *N* = 20 who provided usable data for at least 8 sequences. The respective *N* contributing to each analysis is specified per graph. Significant categorization responses were obtained in all analyses. Averaging across larger numbers of sequences increased response strengths.

Considering the problem of different sample sizes and the trade-off between numbers of sequences and participants who provide sufficient data to be included. In a control analysis with 20 participants who contributed 8 usable sequences, it was evident that increasing numbers of sequences per participant increase both base and categorization responses (Figures 6, 7). This was supported by statistical analyses which showed that the number of sequences included has a significant effect on response strength for the categorization response, *X*^2^(7, *N* = 20) = 17.951, *p* < 0.05. The effect on the base response strength was only marginal, *X*^2^(7, *N* = 20) = 12.651, *p* = 0.081. Bonferroni-corrected pairwise comparisons did not reach significance.

#### Systematically increasing the number of harmonics considered

To evaluate the effect of including harmonics into response measurement, the number of harmonics was systematically increased from 1 to 10 for the base response (corresponding to frequencies 6, 12… 60) and from 1 to 29 for the categorization response (corresponding to frequencies 1.2, 2.4… 34.8, always excluding harmonics corresponding to the base frequency). In addition, a Z-score criterion was used to select the number of harmonics summed as in prior work (base: 1–5, categorization: 1–19).

**Figure 8:**
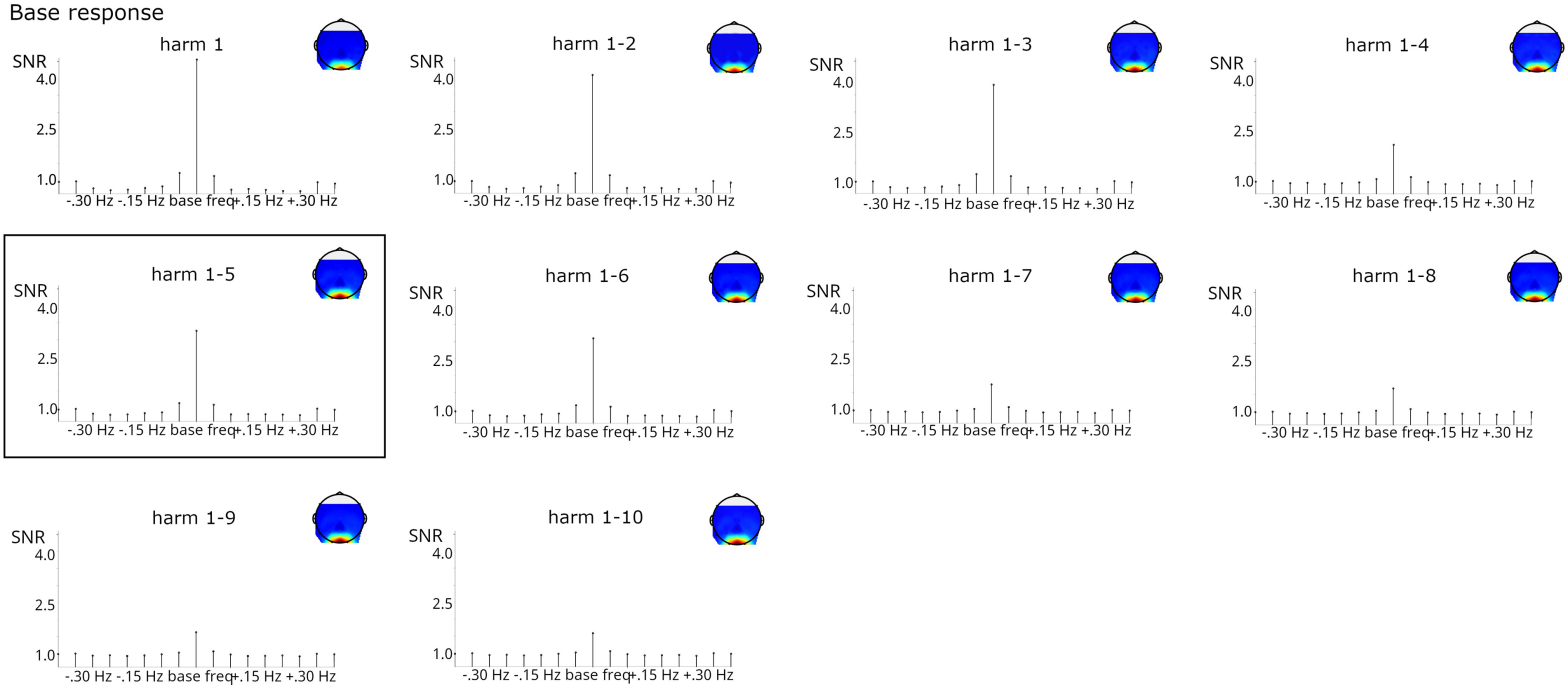
SNR of base grand-average responses summed across increasing numbers of harmonics (considering only the 1^st^ or up to harmonics 1–10). Highly significant responses were obtained in all analyses. The analysis on harmonics 1–5, marked by a black box, was indicated by the Z-score criterion.

As evident from Figure 8, lower base harmonics (1–3) contributed strongly to the summed responses, whereas including higher harmonics (particularly 7–10) descriptively reduced the SNR at the stimulated frequencies. However, estimation of SNR at the surrounding bins employed for baseline correction was also reduced by summing, so overall, including harmonics contributed to a cleaner estimation of base responses. In this case, the number of harmonics for summing obtained by the Z-score criterion, 1–5, provides an optimal balance between high responses at the stimulated frequencies and evenly distributed responses across the surrounding bins. Friedman tests confirmed that the number of harmonics influenced base response strength, *X^2^*(9, *N* = 123) = 182.889, *p* < 0.001. Post-hoc tests indicated that baseline-corrected amplitudes were significantly smaller when considering only the first harmonic (i.e., 6 Hz) compared to analyses including at least 3 harmonics, all *p*s < 0.05. No other comparison reached significance.

**Figure 9:**
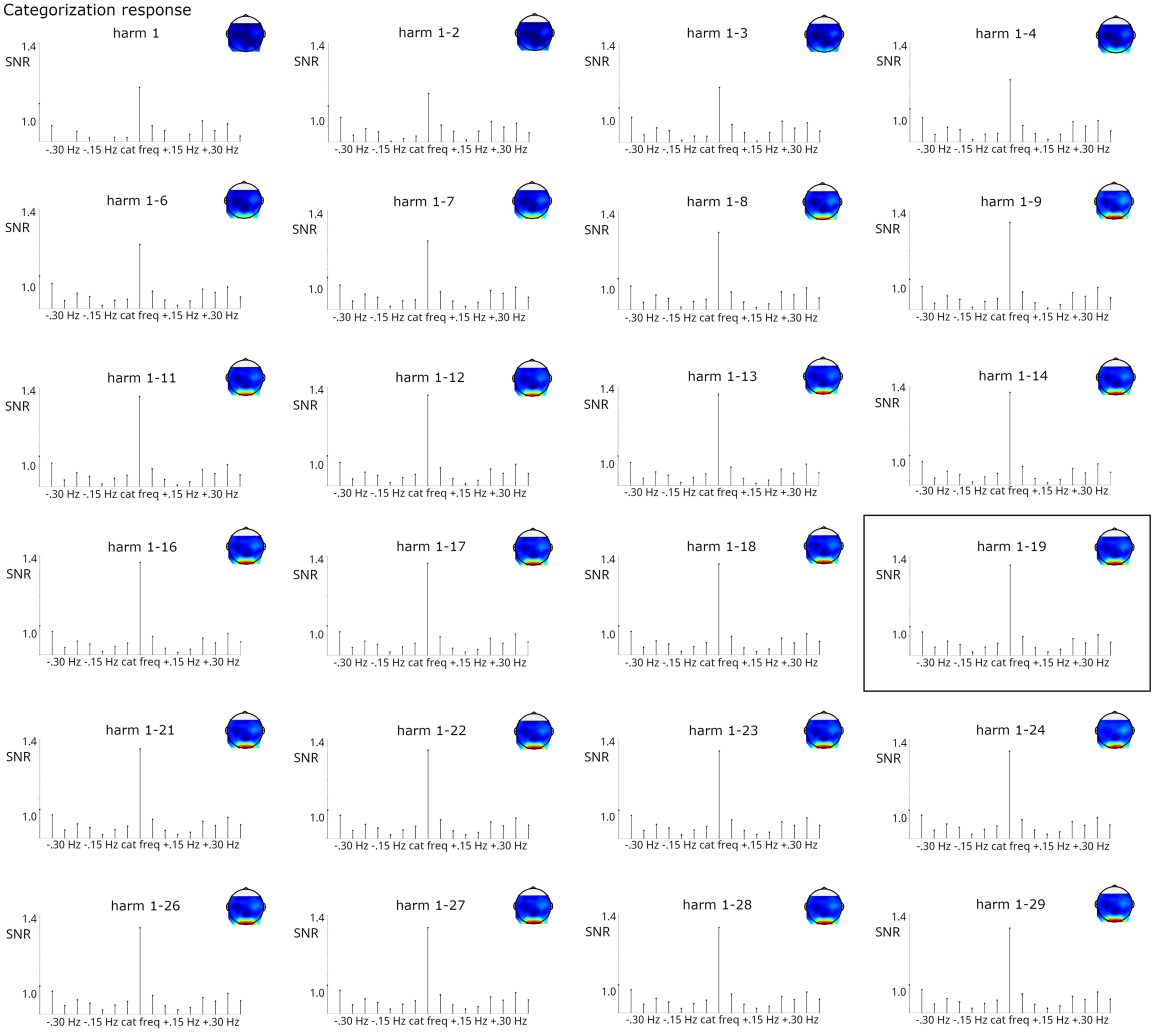
SNR of categorization grand-average responses summed across increasing numbers of harmonics (considering only the 1^st^ or up to harmonics 1–29, always excluding harmonics corresponding to the frequency). Highly significant responses were obtained in all analyses. The analysis on harmonics 1–19, marked by a black box, was indicated by the Z-score criterion.

For the categorization response, including harmonics generally led to an increase in SNR at the stimulated frequencies. As evident from Figure 9, while the categorization response was visible from the 1^st^ harmonic, signal strength increased numerically until summation of harmonics 1–9 and stabilized from that point onward. Signal strength at the surrounding bins was not much influenced beyond harmonics 1–6. Here, including harmonics (at least up to the 9^th^) seems desirable for optimal signal estimation. The analysis based on the Z-score criterion (harmonics 1–19) does not seem to have an advantage in terms of signal at stimulated frequencies or surrounding bins compared to related harmonic ranges (e.g., 1 through 11–1 through 29). For the categorization response, Friedman tests also confirmed that the number of harmonics influenced response strengths, *X^2^*(9, *N* = 123) = 1295.21, *p* < 0.001. Post-hoc tests indicated that baseline-corrected amplitudes were significantly smaller when considering only the first two harmonics (i.e., 1.2 + 2.4 Hz) compared to analyses including at least 3 harmonics, all *p*s < 0.05. Moreover, analyses including at least 16 harmonics yielded higher amplitudes than those including up to 11 harmonics, all *p*s < 0.05.

## Discussion

Based on a systematic literature review and re-analyses of data on categorization in seven-month-old infants, this manuscript evaluates stimulation and analysis decisions of infant frequency tagging studies employing the FPVS oddball paradigm. In the following, current standards and future recommendations will be summarized.

A major challenge in developmental neuroscience is the limited attentional capacity of participants. Prior infant frequency studies following the FPVS oddball paradigm have faced this challenge by reducing sequence duration to 15–30 s. The longer the sequence, the more stimulation can be delivered, and the higher is the spectral resolution of resulting data. Thus, maximizing sequence duration is highly desirable. Given the apparent feasibility of 30-s-sequences, this duration is recommended for future studies.

Relatedly, the number of sequences that can be presented is limited by infants’ attentional capacities. The systematic literature review indicates that on average, about 9 sequences can be presented, so limiting the number of conditions to 2–3 within-subject factors seems reasonable. However, this number will need to be adjusted to the nature of the stimulation and the age of participants, with more engaging presentations (e.g., concurrent visual and auditory stimulation) and older participants being likely to provide more usable data.

Analyzing the effect of the number of sequences included may aid in determining minimum requirements for participant inclusion. Generally, this decision follows a trade-off: The more sequences are set as a requirement for participant retention, the more data is lost due to exclusion of participants. Indeed, increasing the number of required sequences from 1 to 9 led to exclusion of 113 participants. Most important is probably the effect of excluding all participants providing less than two usable sequences. This led to exclusion of 10 participants (8%). However, while the amount of data loss may be considered passable, excluding those participants decreased the categorization response numerically, reflecting the loss of meaningful data. One sequence in the FPVS oddball paradigm displayed at 6 Hz contains, depending on the precise duration, between 90 and 180 images (75–150 base stimuli, 15–30 stimuli from the contrasting category). In addition, generalization within and discrimination across categories is inherent within each sequence and reflected by the categorization response. This argues in favor of including all participants contributing at least one usable sequence to the analysis (or each condition).

Of all presented sequences, several might need to be excluded based on data quality and participant attention. The literature review indicated that data quality was mostly assessed by visual inspection, and data quality issues were treated mainly by exclusion of sequences or channel interpolation. It seems desirable to supplement visual inspection through more automated artifact detection approaches, to increase preprocessing accuracy, objectivity, and efficiency. Certainly this will be an important avenue for future studies.

Sequences with sufficient data quality still need to be screened for attention towards the presentation, as infant attention naturally fluctuates throughout the session. Different approaches have been employed for this step, based on looking times or base rate responses during a given sequence. Comparing such approaches in a re-analysis of a large data-set comprising *N* = 124 seven-month-olds reveals that sequence and participant retention rates are influenced by the criteria employed, whereas the resulting data pattern and the relations of base responses with looking time do not vary much. To maximize data retention and in accord with most prior studies, use of an SNR (>2) or Z-score (>2.33) criterion based on only one channel (O1, O2, or Oz) can thus be recommended.

Due to the shorter sequence duration employed with developmental populations, the frequency resolution of resulting data is decreased. This should be taken into consideration when estimating the signal at each bin and may be especially important in the low frequency spectrum, which is typically characterized by a larger amount of noise and distortions due to the FFT. Thus, employing a large range of bins for noise estimation, e.g., to determine Z-scores or SNRs, may disproportionately affect responses in the low spectrum, such as 1.2 Hz. This was reflected in the re-analysis reported here, which showed a decrease in response strength at lower frequencies (<6 Hz), but an increase of responses at higher frequencies (>6 Hz) when employing 20 bins for baseline correction. It is therefore recommended to employ a reduced frequency range (e.g., ~ + −0.35 Hz, corresponding to 10 bins) when stimulating for up to 30 s.

Two final decisions that should be made *a priori* regard the selection of harmonics for summing and electrodes for analysis. Due to the limited number of studies comparing infant responses in the same paradigm to that of older participants, recommendations are based only on anecdotal evidence at this point. It seems likely that responses can be confined to fewer harmonics and electrodes early in life than later on, so simply basing these decisions on adult data does not seem appropriate. Therefore, similar, *a priori* defined criteria should be employed to select electrodes and harmonics across age-groups. For harmonics, a selection based on Bonferroni-corrected significance levels of harmonics can be recommended (Retter et al., 2021). This selection seemed optimal in the analysis of base responses. In the analysis of categorization responses, while summing across several harmonics clearly had advantages in terms of signal strength at stimulated and surrounding frequencies, none of the analyses could be clearly favored, as the responses remained relatively stable from summing harmonics 1–11 onward. In such a case, considering Z-scores may provide an objective criterion for deciding which harmonics to include.

For electrodes, decisions are generally based more on theoretical accounts and prior studies but should be open to adjustments based on age-group. In any case, transparent and *a priori* selection is advisable, for example through preregistering analyses.

### Limitations

Given the particularities of the approach, the systematic review was limited to studies employing the FPVS oddball paradigm with infants, so this overview and the recommendations derived are likewise limited. However, they may be useful for other developmental frequency tagging studies following related stimulation paradigms.

Moreover, the data re-analysis was based on only one age-group (seven-month-olds), and a categorization paradigm with artificial, unfamiliar stimuli that should not evoke much prior experience. Accordingly, categorization responses were elicited primarily in the occipital region. In prior studies using more familiar categories, responses were sometimes also recorded in the occipital region (Peykarjou et al., under review see footnote 1; Barry-Anwar et al., 2018), but in other cases at more parietal and/or temporal regions (de Heering and Rossion, 2015; Leleu et al., 2020). Future work should certainly test and generalize analysis recommendations to categorization tasks involving other regions of interest. Moreover, the categorization response elicited in the present study is much stronger than in most studies using familiar, natural categories (Baccolo et al., in preparation see footnote 3; De Heering and Rossion, 2015; Barry-Anwar et al., 2018; Leleu et al., 2020; Rekow et al., 2021). Upon investigating a subtler response, it seems likely that analysis decisions will carry even more weight than for the robust response observed in the current paradigm.

While these limitations may of course restrict generalizability, most analysis decisions evaluated here should in principle be independent of the specific age-group and stimuli presented. The increased noise level in the low frequency spectrum will be evident across infant samples of all research questions, arguing in favor of generally employing 10 bins for baseline correction. While sequence and participant retention rates will vary with age-group and stimulation, the general pattern of being more inclusive (and thus representative) when employing a base-rate criterion directed at only one occipital channel will likely be independent of these aspects.

In contrast, of course the response patterns resulting from different sequence inclusion criteria may vary. On the one hand, there is no indication to assume that being less inclusive (i.e., requiring base rate responses on more channels, or excluding participants providing less than one usable sequence) will lead to enhanced data cleanness. On the other hand, keeping more data will increase the representativeness of analyses for the tested sample. The observation that the number of sequences included did, overall, not change base or categorization data patterns much likely reflects the overall amount of data analyzed. It seems probable that the effects are less driven by the number of sequences *per se* but rather the overall amount of data (i.e., participants * sequences), which again argues for an inclusive criterion. However, his question should also be addressed in future studies, as it is likely not independent from study-specific factors.

### Open questions and avenues

As we are beginning to systematically develop and evaluate developmental frequency tagging, some central aspects remain to be addressed in future work. First, as already mentioned in the Introduction, frequency sweeps testing the optimal flicker speed with infant participants are lacking. These would be highly desirable not only to optimize the stimulation of developmental frequency tagging, but also to further our understanding of the development of basic neural processes. For instance, certain frequency bands have been associated with particular cognitive functions (e.g., theta range: ~ 4 Hz in infants; learning; e.g., Begus et al., 2015, alpha range: ~ 6 Hz in infants; attention; e.g., Friese et al., 2013; Gulbinaite et al., 2019). Labeling frequency bands in a consistent way across development is challenging, and more basic research is needed to ascertain the relations between cognitive functions and given frequencies at different ages. Moreover, a systematic analysis of the assumption that stimulating at certain frequencies supports particular cognitive processes would require sampling a large range of frequencies. Inherently, stimulating at a given frequency also means presenting stimuli for a certain period of time, and changing presentation times may in itself alter cognitive processes. Therefore, systematically varying the speed of stimulation and associating it with response patterns and cognitive functions seems an important avenue for future work.

Moreover, to be able to apply frequency tagging more broadly, reliability of responses needs to be evaluated. The promising reliabilities obtained in adult face individuation studies (Dzhelyova et al., 2019; Stacchi et al., 2019) give rise to the hope that frequency tagging may provide a window into cognitive processes that is suited even for differential approaches and longitudinal research. Both within-and between-sessions reliability of base and categorization responses should be examined to ascertain the utility of the approach. While relatively long inter-session intervals (2–6 months) have been employed with adults, developmental progress makes it unlikely that responses will remain stable over such time-windows. However, frequency tagging seems to have the potential to provide reliable estimates of categorization, at least on the time-scale of a few weeks.

More broadly speaking, the paradigm holds potential for investigating cognitive processes beyond categorization. For example, it might be useful for investigating rule learning, the process of extracting and generalizing repetition-based, abstract patterns to new elements (Marcus et al., 1999). Moreover, it could be used concurrently (and even orthogonally) in other tasks to track attention, when a cognitive task is presented in the context of an object or background flickering at a given frequency. The relation between frequency tagging responses and attention has been broadly established in studies with adult participants (Morgan et al., 1996; Chen et al., 2003; Toffanin et al., 2009). Similarly, the relation of infant frequency tagging responses to overt and covert visual attention has been established previously (Christodoulou et al., 2018), but the approach has so far not been used as a control mechanism during an independent task. The dissociated and objective measure of the base rate response could become a valuable tool in developmental cognitive neuroscience.

## Conclusion

Frequency tagging is a tool increasingly utilized in developmental cognitive neuroscience and has previously been employed to demonstrate fast and high-level face and object categorization in the first year of life (De Heering and Rossion, 2015; Kabdebon et al., 2022; Peykarjou et al., under review See footnote 1). Objectivity and a high signal-to-noise ratio are inherent to this approach, and its reliability and validity have been demonstrated in adult research. This paper has provided a systematic review of studies following the FPVS oddball paradigm and a re-analysis of a dataset comprising 124 seven-month-old infants to develop recommendations for future work following this approach. Future work should set a particular focus on automizing and objectifying artifact detection, assessing the reliability of infant frequency tagging responses, and systematically evaluating the development of infants’ response patterns across a range of frequencies. Together, these endeavors will serve to enhance the usefulness of frequency tagging for research on cognitive development, in basic functions such as categorization and beyond.

## Data availability statement

The original contributions presented in the study are included in the article/supplementary material, further inquiries can be directed to the corresponding author.

## Ethics statement

The studies involving human participants were reviewed and approved by Ethikkommission Fakultät für Verhaltens- and Empirische Kulturwissenschaften, Universität Heidelberg. Written informed consent to participate in this study was provided by the participants’ legal guardian/next of kin.

## Author contributions

SP conceptualized the manuscript, performed analyses, and wrote the manuscript.

## Conflict of interest

The author declares that the research was conducted in the absence of any commercial or financial relationships that could be construed as a potential conflict of interest.

## Publisher’s note

All claims expressed in this article are solely those of the authors and do not necessarily represent those of their affiliated organizations, or those of the publisher, the editors and the reviewers. Any product that may be evaluated in this article, or claim that may be made by its manufacturer, is not guaranteed or endorsed by the publisher.

## Footnotes

^4^Of course, this assumes that adequate corrections for multiple tests across harmonics have been adopted.

^5^Pauen, S. and Peykarjou, S. (under revision). Infants’ Fast Neural Categorization of Artificial Objects: The impact of stimulus- and task- characteristics Developmental Psychology.

^6^ https://nocions.github.io/letswave6/
